# Supplementary figures and images for: Psychological distress in late adolescence: The role of inequalities in family affluence and municipal socioeconomic characteristics in Norway
Source: PLoS One. 2021 Jul 2;16(7):e0254033. doi: 10.1371/journal.pone.0254033 (PMC8253448; doi:10.1371/journal.pone.0254033)

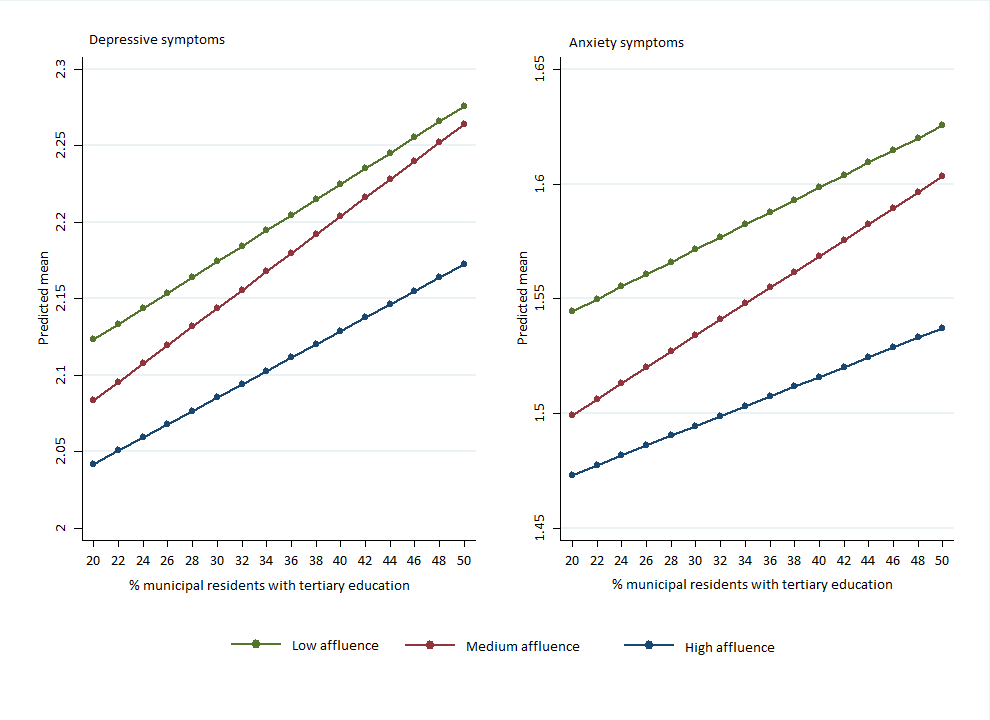

Supplement: S1 Fig — (TIF) [file pone.0254033.s001.tif]

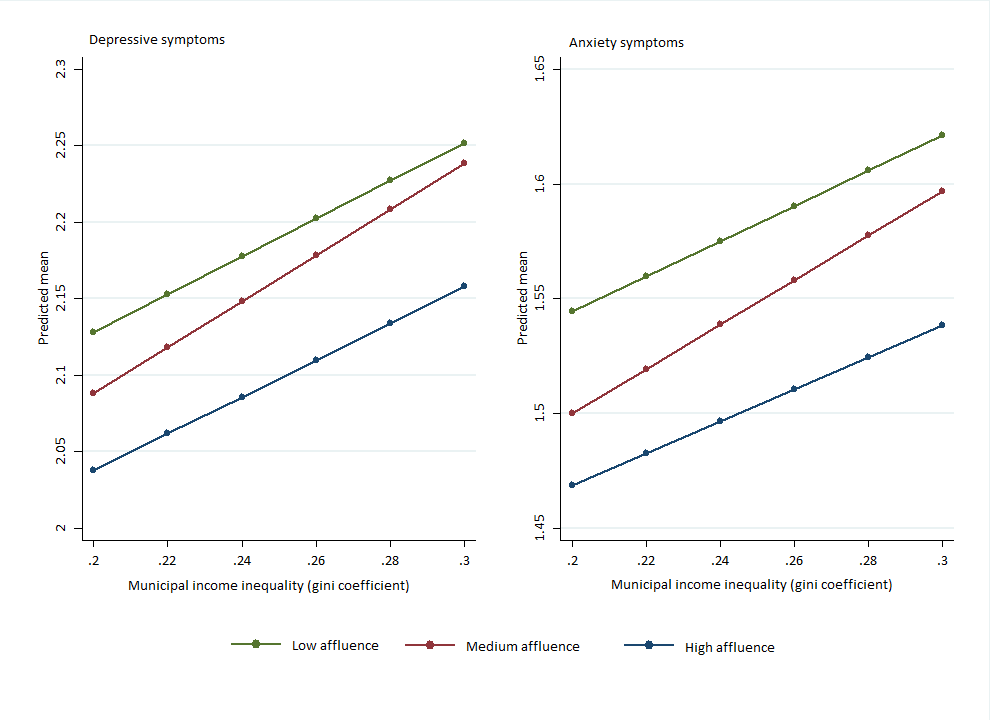

Supplement: S2 Fig — (TIF) [file pone.0254033.s002.tif]
