# Supplementary material for: Psychological distress in late adolescence: The role of inequalities in family affluence and municipal socioeconomic characteristics in Norway
Source: PLoS One. 2021 Jul 2;16(7):e0254033. doi: 10.1371/journal.pone.0254033 (PMC8253448; doi:10.1371/journal.pone.0254033)
Supplement: S1 Table — (DOCX) [file pone.0254033.s003.docx]

|  | **Psychological symptoms** | **Depressive symptoms** | **Anxiety symptoms** |
| --- | --- | --- | --- |
|  | OR (95 % CI) | OR (95 % CI) | OR (95 % CI) |
| **Fixed components** |  |  |  |
| Constant | 0.27 (0.26 to 0.29) | 0.44 (0.41 to 0.46) | 0.07 (0.07 to 0.08) |
| **Individual level** |  |  |  |
| Gender (female) | 3.76 (3.61 to 3.91) | 3.30 (3.17 to 3.44) | 4.15 (3.95 to 4.36) |
| Family affluence |  |  |  |
| High | Ref |  |  |
| Medium | 1.18 (1.14 to 1.22) | 1.16 (1.12 to 1.20) | 1.18 (1.13 to 1.22) |
| Low | 1.26 (1.22 to 1.31) | 1.20 (1.17 to 1.25) | 1.34 (1.30 to 1.40) |
| School year |  |  |  |
| Year 1 | Ref |  |  |
| Year 2 | 1.04 (0.99 to 1.09) | 1.04 (1.00 to 1.09) | 1.02 (0.96 to 1.08) |
| Year 3 | 1.41 (1.34 to 1.49) | 1.43 (1.36 to 1.50) | 1.18 (1.10 to 1.26) |
| Gender x school year |  |  |  |
| Female x year 1 | Ref |  |  |
| Female x year 2 | 0.96 (0.91 to 1.03) | 0.99 (0.93 to 1.05) | 0.89 (0.82 to 0.96) |
| Female x year 3 | 0.81 (0.75 to 0.87) | 0.88 (0.82 to 0.94) | 0.77 (0.71 to 0.84) |
| Survey cycle | 1.14 (1.12 to 1.15) | 1.07 (1.06 to 1.08) | 1.14 (1.13 to 1.15) |
| **Random components** |  |  |  |
| sd (cons) | 0.04 (0.03 to 0.06) | 0.04 (0.03 to 0.06) | 0.02 (0.02 to 0.04) |
| ICC municipality (%) | 1.22 | 1.20 | 0.73 |
| AIC | 122756.4 | 124242.5 | 101678.7 |
| BIC | 122851.2 | 124337.4 | 101773.6 |

**S1 Table.** Odds ratios (ORs) of moderate-to-high psychological symptoms by use of multilevel logistic regression model
