# Supplementary material for: Psychological distress in late adolescence: The role of inequalities in family affluence and municipal socioeconomic characteristics in Norway
Source: PLoS One. 2021 Jul 2;16(7):e0254033. doi: 10.1371/journal.pone.0254033 (PMC8253448; doi:10.1371/journal.pone.0254033)
Supplement: S2 Table — (DOCX) [file pone.0254033.s004.docx]

**S2 Table.** The impact of family affluence, municipal education level and their interactions* for risk of moderate to high levels of psychological distress, depressive and anxiety symptoms among high school students in Norway.

|  | **Psychological symptoms** | | **Depressive symptoms** | | **Anxiety symptoms** | |
| --- | --- | --- | --- | --- | --- | --- |
|  | Main effect model | Interaction model | Main effect model | Interaction model | Main effect model | Interaction model |
|  | OR (95% CI) | OR (95% CI) | OR (95% CI) | OR (95% CI) | OR (95% CI) | OR (95% CI) |
| **Fixed effects** |  |  |  |  |  |  |
| **Individual level** |  |  |  |  |  |  |
| Family affluence |  |  |  |  |  |  |
| High | Ref | Ref | Ref | Ref | Ref | Ref |
| Medium | 1.18  (1.14-1.22) | 1.06  (0.95-1.18) | 1.17  (1.13-1.21) | 1.03  (0.92-1.14) | 1.18  (1.14-1.23) | 1.05  (0.93-1.19) |
| Low | 1.27  (1.23-1.31) | 1.26  (1.13-1.41) | 1.21  (1.17-1.25) | 1.23  (1.10-1.37) | 1.35  (1.30-1.40) | 1.24  (1.10-1.41) |
| **Municipal level** |  |  |  |  |  |  |
| % tertiary education | 1.01  (1.01-1.02) | 1.01  (1.01-1.02) | 1.01  (1.01-1.02) | 1.01  (1.01-1.02) | 1.01  (1.01-1.01) | 1.01  (1.003-1.01) |
| **Cross-level interactions** |  |  |  |  |  |  |
| Family affluence x % tertiary education | |  |  |  |  |  |
| High x education |  | Ref |  |  |  |  |
| Medium x education |  | 1.003  (1.0002-1.01) |  | 1.004  (1.001-1.01) |  | 1.003  (1.00-1.01) |
| Low x education |  | 1.0001  (0.997-1.00) |  | 1.00  (1.00-1.00) |  | 1.002  (1.00-1.01) |
| **Random effects** |  |  |  |  |  |  |
| At level 2: intercept | 0.03  (0.02-0.04) | 0.03  (0.02-0.04) | 0.03  (0.02-0.04) | 0.03  (0.02-0.04) | 0.02  (0.01-0.03) | 0.02  (0.01-0.03) |
| ICC (%) | 0.94 | 0.94 | 0.93 | 0.92 | 0.57 | 0.56 |
| AIC | 122733.1 | 122731.7 | 124222 | 124217.1 | 101661.5 | 101661.6 |
| BIC | 122837.4 | 122854.9 | 124326.3 | 124340.5 | 101765.8 | 101784.9 |
