# Supplementary material for: Psychological distress in late adolescence: The role of inequalities in family affluence and municipal socioeconomic characteristics in Norway
Source: PLoS One. 2021 Jul 2;16(7):e0254033. doi: 10.1371/journal.pone.0254033 (PMC8253448; doi:10.1371/journal.pone.0254033)
Supplement: S3 Table — (DOCX) [file pone.0254033.s005.docx]

**S3 Table.** The impact of family affluence, municipal income inequality (gini-coefficient) and their interactions* for risk of moderate to high levels of psychological distress, depressive and anxiety symptoms among high school students in Norway.

|  | **Psychological symptoms** | | **Depressive symptoms** | | **Anxiety symptoms** | |
| --- | --- | --- | --- | --- | --- | --- |
|  | Main effect model | Interaction model | Main effect model | Interaction model | Main effect model | Interaction model |
|  | OR (95% CI) | OR (95% CI) | OR (95% CI) | OR (95% CI) | OR (95% CI) | OR (95% CI) |
| **Fixed effects** |  |  |  |  |  |  |
| **Individual level** |  |  |  |  |  |  |
| Family affluence |  |  |  |  |  |  |
| High | Ref | Ref | Ref | Ref | Ref | Ref |
| Medium | 1.18 (1.14-1.22) | 1.06 (0.88-1.27) | 1.16 (1.13-1.20) | 0.97 (0.81-1.17) | 1.18 (1.14-1.23) | 1.08 (0.87-1.33) |
| Low | 1.27 (1.22-1.31) | 1.34(1.12-1.61) | 1.21 (1.17-1.25) | 1.34 (1.12-1.61) | 1.35 (1.30-1.40) | 1.31 (1.07-1.60) |
| **Municipal level** |  |  |  |  |  |  |
| Income inequality (gini) | 18.83  (5.42-65.42) | 17.94  (4.86-66.19) | 30.76  (9.04-104.68) | 29.07  (8.04-105.18) | 20.66  (6.71-63.63) | 17.71  (5.27-59.54) |
| **Cross-level interactions** |  |  |  |  |  |  |
| Family affluence x income inequality (gini) | |  |  |  |  |  |
| High x income inequality |  | Ref |  | Ref |  | Ref |
| Medium x income inequality |  | 1.55 (0.76-3.15) |  | 2.04 (1.00-4.15) |  | 1.44 (0.65-3.19) |
| Low x income inequality |  | 0.80 (0.40-1.60) |  | 0.65 (0.32-1.30) |  | 1.12 (0.52-2.43) |
| **Random effects** |  |  |  |  |  |  |
| At level 2: intercept | 0.04(0.03-0.05) | 0.04 (0.03-0.05) | 0.03 (0.02-0.05) | 0.03 (0.02-0.05) | 0.02(0.01-0.03) | 0.02 (0.01-0.03) |
| ICC (%) | 1.05 | 1.05 | 1.00 | 1.00 | 0.60 | 0.59 |
| AIC | 122738 | 122738.8 | 124215.6 | 124210.5 | 101653.4 | 101656.5 |
| BIC | 122842.2 | 122862.1 | 124319.9 | 124333.9 | 101757.7 | 101779.8 |
